# Buoyancy-modulated Lagrangian drift in wavy-walled vertical channels as a model problem to understand drug dispersion in the spinal canal

**DOI:** 10.1017/jfm.2022.799

**Published:** 2022-10-06

**Authors:** J. Alaminos-Quesada, W. Coenen, C. Gutiérrez-Montes, A.L. Sánchez

**Affiliations:** 1 Department of Mechanical and Aerospace Engineering, University of California San Diego, La Jolla, CA 92093-0411, USA; 2 Grupo de Mecánica de Fluidos, Universidad Carlos III de Madrid, Leganés 28911, Spain; 3 Andalusian Institute for Earth System Research, University of Jaén, Jaén 23071, Spain

**Keywords:** biomedical flows

## Abstract

This paper investigates flow and transport in a slender wavy-walled vertical channel subject to a prescribed oscillatory pressure difference between its ends. When the ratio of the stroke length of the pulsatile flow to the channel wavelength is small, the resulting flow velocity is known to include a slow steady-streaming component resulting from the effect of the convective acceleration. Our study considers the additional effect of gravitational forces in configurations with a non-uniform density distribution. Specific attention is given to the slowly evolving buoyancy-modulated flow emerging after the deposition of a finite amount of solute whose density is different from that of the fluid contained in the channel, a relevant problem in connection with drug dispersion in intrathecal drug delivery (ITDD) processes, involving the injection of the drug into the cerebrospinal fluid that fills the spinal canal. It is shown that when the Richardson number is of order unity, the relevant limit in ITDD applications, the resulting buoyancy-induced velocities are comparable to those of steady streaming. As a consequence, the slow time-averaged Lagrangian motion of the fluid, involving the sum of the Stokes drift and the time-averaged Eulerian velocity, is intimately coupled with the transport of the solute, resulting in a slowly evolving problem that can be treated with two-time-scale methods. The asymptotic development leads to a time-averaged, nonlinear integro-differential transport equation that describes the slow dispersion of the solute, thereby circumventing the need to describe the small concentration fluctuations associated with the fast oscillatory motion. The ideas presented here can find application in developing reduced models for future quantitative analyses of drug dispersion in the spinal canal.

## 1. Introduction

The steady Lagrangian drift generated in oscillatory viscous flows in pipes and channels is known to play an important role in different heat and mass transport processes, including those occurring in extracorporeal membrane oxygenators (Bellhouse *et al*. 1973), pulmonary high-frequency ventilation devices (Grotberg 1994), compact heat exchangers (Mackley & Stonestreet 1995) and drug dispersion in the spinal canal (Lawrence *et al*. 2019). For configurations with slowly varying cross-section, the lubrication approximation can be used to derive insightful analytical results, with seminal analyses including those of Hall (1974), who considered flow in a pipe subject to a harmonic pressure difference, and Grotberg (1984), who investigated flow in a tapered channel subject to a prescribed oscillating stroke volume. More recent analytical studies pertaining to channels include those of Lo Jacono, Plouraboué & Bergeon (2005) and Guibert, Plouraboué & Bergeon (2010), involving three-dimensional wavy-walled configurations, and that of Larrieu, Hinch & Charru (2009), who considered an oscillating Couette flow over a wavy bottom. All of these analytical investigations of oscillating slender flows addressed configurations with weak inertia, corresponding to small values of the ratio ε of the stroke length to the characteristic longitudinal length, with $\varepsilon^{-1}\gg 1$ representing the relevant Strouhal number. The asymptotic analysis for ε≪1 reveals that the velocity, resulting from a balance between the local acceleration and the pressure and viscous forces, is harmonic at leading order, with the small corrections arising from the convective acceleration providing a small steady-streaming component of order ε (Riley 2001). This steady streaming determines, together with the Stokes drift associated with the leading-order harmonic flow, the Lagrangian drift experienced by the fluid particles, with both contributions having in general comparable orders of magnitude (Larrieu *et al*. 2009).

**Figure 1.**
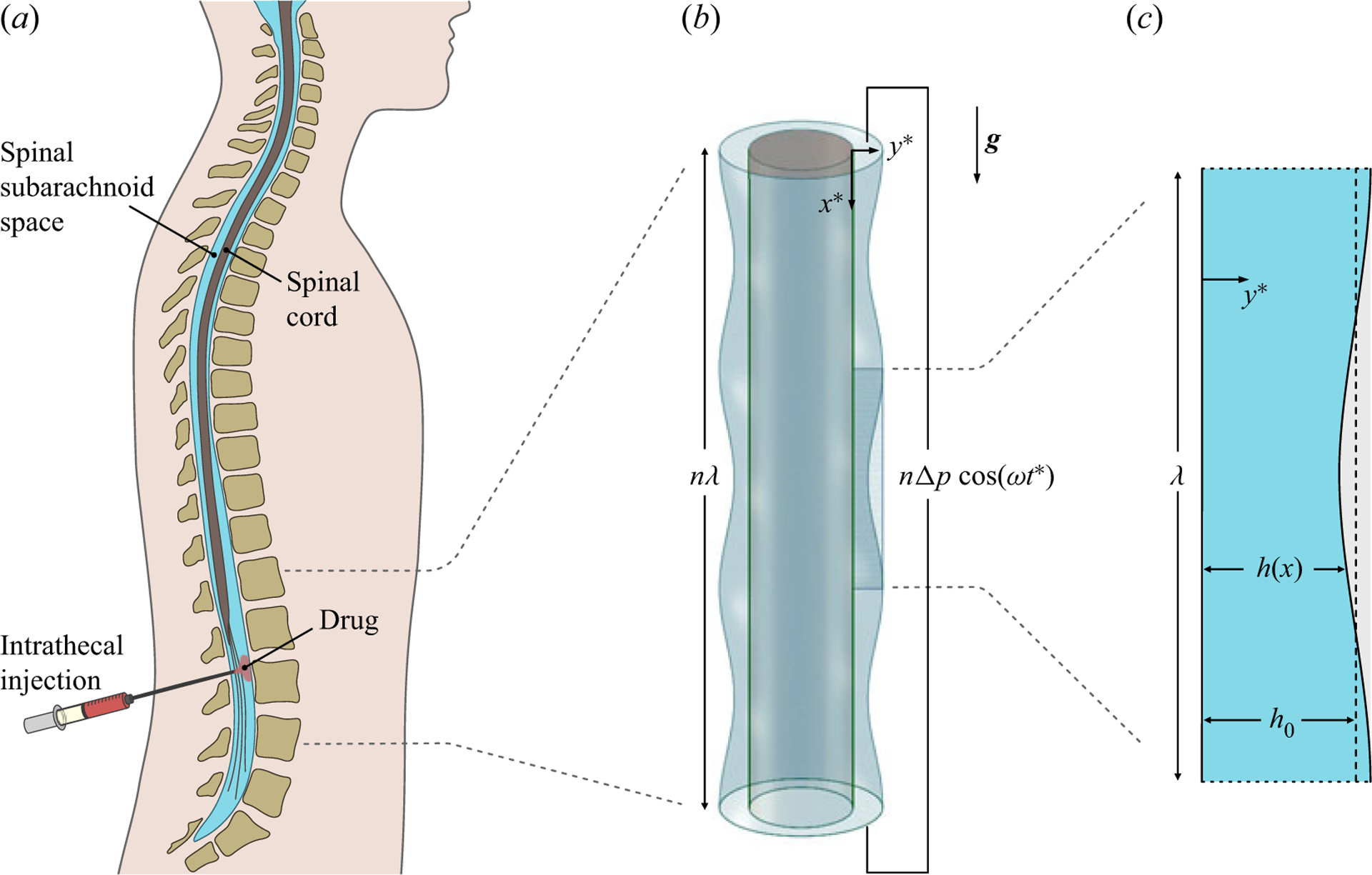
A schematic view of the spinal canal, showing the location of (*a*) ITDD and (*b, c*) of the two-dimensional channel flow investigated here.

As discussed by Guibert *et al*. (2010), the fundamental pulsatile-flow investigations mentioned previously are relevant in connection with the motion of cerebrospinal fluid (CSF) along the spinal subarachnoid space, a slender annular canal surrounding the spinal cord, depicted in figure 1. The flow features an oscillatory velocity driven by the pressure pulsations induced by the cardiac and respiratory cycles (Linninger *et al*. 2016). The dynamics of this flow and its associated Lagrangian transport are fundamental in understanding the role of CSF as a vehicle for metabolic-waste clearance (Klarica, Radoš & Orešković 2019) and also to quantify drug dispersion in intrathecal drug delivery (ITDD) (Onofrio 1981; Lynch 2014; Fowler *et al*. 2020), a medical procedure used for treatment of some cancers (Lee *et al*. 2017), infections (Remeš *et al*. 2013) and pain (Bottros & Christo 2014). The standard ITDD protocol involves the placement of a small catheter along the lumbar section of the spinal canal to continuously pump the drug or to release a finite dose at selected times. The transport of the drug depends fundamentally on its physical properties, including molecular diffusivities κ that are much smaller than the kinematic viscosity v of CSF.

The flow in the spinal canal has been investigated computationally in numerous studies addressing different aspects of the problem, as summarised in a recent paper by Khani *et al*. (2018), although corresponding theoretical analyses are more scarce. Exact solutions for pulsatile viscous flow in a straight elliptic annulus have been proposed as a representation for the oscillatory flow in the spinal canal (Gupta, Poulikakos & Kurtcuoglu 2008). More recent studies, modelling the canal as a linearly elastic annular pipe of slowly varying section, have employed the lubrication approximation in the asymptotic limit ε≪1 to quantify steady streaming (Sánchez *et al*. 2018) and to investigate the dispersion of a solute (Lawrence *et al*. 2019). For the large values of the Schmidt number v/κ~1000 corresponding to the molecular diffusivities of all ITDD drugs, the analysis of Lawrence *et al*. (2019) showed that the Lagrangian mean flow is the key mechanism responsible for the dispersion of the solute, whereas shear-enhanced Taylor dispersion has a negligibly small effect. An important outcome of the asymptotic analysis is a time-averaged transport equation that has been recently validated by means of comparisons with results of direct numerical simulations (DNS) spanning hundreds of oscillation cycles (Gutiérrez-Montes *et al*. 2021), as needed to generate significant dispersion of the solute. The comparisons demonstrate the accuracy of the reduced description, which is seen to provide excellent fidelity at a fraction of the computational cost involved in the DNS.

Our previous analysis of solute dispersion (Lawrence *et al*. 2019) assumed the density of the solute $\rho_{s}$ and the density of the carrier fluid ρ to be identical, thereby neglecting the small density differences $\rho -\rho_{S}$ found in ITDD applications, for which the values of $\rho -\rho_{s}$ typically range from positive values of order $\left(\rho -\rho_{s}\right)\sim \rho /1000$ for drugs diluted with water to negative values of order $\left(\rho -\rho_{s}\right)/\sim -\rho /100$ for drugs diluted with dextrose (Nicol & Holdcroft 1992; Lui, Polis & Cicutti 1998). These relative density differences $\left(\rho -\rho_{s}\right)/\rho \ll 1$ between the drug and the CSF, although extremely small, are known by clinicians to play an important role in the dispersion rate of ITDD drugs for patients in a sitting position, when buoyancy forces are nearly aligned with the canal. It has been seen that for a *hyperbaric* (dense) solution, injection while the patient is seated for some time before moving to a supine position leads to an initial restriction in the transport of the anaesthesia (Mitchell *et al*. 1988; Povey, Jacobsen & Westergaard-Nielsen 1989; Veering *et al*. 2001). (In spinal anaesthesia, *baricity* is the term used to refer to the density of the anaesthetic relative to that of the CSF. Thus, an anaesthetic is said to be *hyperbaric/hypobaric* when its density is higher/lower than that of the CSF, whereas the term isobaric describes anaesthetics whose density matches exactly that of the CSF.) Conversely, for a *hypobaric* (light) solution, the sitting injection position leads to more rapid cephalad spread of the anaesthesia as compared to a lateral injection position (Richardson *et al*. 1996). As could be expected, the density of the drug is inconsequential when injection occurs in the lateral position (Hallworth *et al*. 2005) and, similarly, positioning has no effect on the spread rate when the solution density matches that of CSF (Wildsmith *et al*. 1981). Given the abundance of clinical evidence on the importance of buoyancy forces on the drug dispersion rate, there is interest in developing a quantitative description; the present paper, focused on a simplified geometry, is a necessary first step in that direction.

In looking for a simplified geometrical model, we follow Guibert *et al*. (2010) in noting that the width $h_{o}\sim 1-2\;\text{mm}$ of the annular spinal canal is smaller than the spinal-cord diameter ~1cm, with the consequence that a two-dimensional channel can be used to describe many aspects of the flow. The channel is placed in a vertical position, as is appropriate in describing buoyancy effects for a patient in a sitting position. As indicated in figure 1, the quasi-periodic variation of the canal section, associated with the presence of the vertebrae, will be modelled by including a wavy boundary whose wavelength λ mimics the inter-vertebral distance. The channel will be assumed to be slender in that $\lambda \gg h_{o}$, a good approximation in the spinal canal, where λ~2−4cm and $h_{o}/\lambda ≃0.05$. For simplicity, the total channel length is taken to be an integer multiple of the wavelength, so that the channel contains a finite number of identical cells. As in the seminal analysis of Hall (1974), an oscillating pressure difference with angular frequency ω will be imposed between the channel ends, resulting in a pulsating flow. We investigate the buoyancy-modulated dispersion of a bolus of solute released inside the channel when the buoyancy-induced acceleration is comparable to the convective acceleration of the pressure-driven flow, those being the conditions of interest in ITDD applications, as explained later below (2.6).

The rest of the paper is organised as follows. The problem is formulated in dimensionless form in §2, which includes the identification of the relevant non-dimensional parameters and a discussion of the essential features of the subsequent asymptotic analysis, including the existence of a long time scale $\varepsilon^{-2}\omega^{-1}$ for solute dispersion, additional to the much smaller oscillation time $\omega^{-1}$. The asymptotic description of the velocity field is presented next in §3, with the time-averaged Eulerian velocity including the familiar steady-streaming contribution stemming from the convective acceleration along with an additional buoyancy-induced component that depends on the distribution of solute. This velocity field is used in §4 to analyse solute dispersion with use of a two-time scale asymptotic analysis, resulting in a time-averaged transport equation that describes the evolution of the flow in the long-time scale $\varepsilon^{-2}\omega^{-1}$. The reduced description stemming from the asymptotic analysis is validated in §5 through comparisons with DNS. In addition, the model is used to quantify effects of buoyancy-induced motion on the solute dispersion for different values of the controlling parameters. Finally, concluding remarks are given in §6.

## 2. Problem formulation

### 2.1. Governing equations

Consider a vertical wavy channel of average gap size $h_{o}$ filled with a Newtonian fluid of density ρ and kinematic viscosity v (for CSF, $\rho ≃10^{3}\;\text{kg}{\;\text{m}}^{-3}$ and $v≃0.7\times 10^{-6}{\;\text{m}}^{2}{\;\text{s}}^{-1}$). The channel, open at both ends, is bounded by a flat surface and a wavy wall of wave length $\lambda \gg h_{o}$, so that the resulting channel width is $h=h_{o}\left[1+\beta \text{cos}\;\left(2\pi x^{*}/\lambda \right)\right]$, where $x^{*}$ is the longitudinal distance measured from the upper end and β<1 is the relative amplitude of the wall undulation. The total channel length is nλ, with n representing a general integer number, so that the channel comprises n identical cells. The flow is described using cartesian coordinates $\left(x^{*},y^{*}\right)$, with $y^{*}$ measured from the flat surface, and corresponding velocity components $v^{*}=\left(u^{*},v^{*}\right)$. The Navier–Stokes equations describing the planar unsteady flow are written in the Boussinesq approximation

(2.1) $$\nabla^{*}\cdot v^{*}=0,$$

(2.2) $$\frac{\partial v^{*}}{\partial t^{*}}+v^{*}\cdot \nabla^{*}v^{*}=-\frac{1}{\rho }\nabla^{*}p^{*}+\nu \nabla^{*2}v^{*}-\frac{\rho -\rho_{s}}{\rho }gce_{x},$$

(2.3) $$\frac{\partial c}{\partial t^{*}}+v^{*}\cdot \nabla^{*}c=\kappa \nabla^{*2}c,$$

where $p^{*}$ is the sum of the pressure difference from the upper end and the constant-density hydrostatic component $-\rho gx^{*},c$ is the solute volume concentration, κ is the solute diffusivity, $\nabla^{*}=\left(\partial /\partial x^{*},\partial /\partial y^{*}\right)$ and $e_{x}$ is the unit vector aligned with the gravitational acceleration.

A pressure difference $n\Delta p\text{cos}\;\left(\omega t^{*}\right)$ oscillating harmonically in time is prescribed between the upper and lower ends of the canal, driving a periodic fluid motion with angular frequency ω. The resulting slender flow is characterised by longitudinal velocities of order $u_{c}=\Delta p/(\rho \omega \lambda )$, as follows from a balance between the local acceleration $\partial u^{*}/\partial t^{*}\sim u_{c}\omega$ and the pressure gradient $\rho^{-1}\partial p^{*}/\partial x^{*}\sim \Delta p/(\lambda \rho )$, and much smaller transverse velocities of order $v_{c}=\left(h_{o}/\lambda \right)u_{c}\ll u_{c}$, as follows from the continuity balance $\partial u^{*}/\partial x^{*}\sim \partial v^{*}/\partial y^{*}$.

### 2.2. Controlling parameters

The analysis assumes that the viscous time across the channel $h_{o}^{2}/v$ is comparable to the characteristic oscillation time $\omega^{-1}$, resulting in Womersley numbers

(2.4) $$\alpha ={\left(\frac{\omega h_{o}^{2}}{v}\right)}^{1/2},$$

of order unity. The limit α~1 is instrumental in analysing cardiac-driven CSF flow $\left(\omega =2\pi {\text{s}}^{-1}\right)$ in the spinal canal, for which typical values of α are in the range 3≲α≲6, as can be seen by evaluating the above expression with $h_{o}≃1-2\;\text{mm}$ and $v=0.7\times 10^{-6}{\;m}^{2}{\;s}^{-1}$. In the lumbar region, the typical drug-delivery site in ITDD procedures, the average CSF speeds are of order $u_{c}\sim 1\;cm{\;s}^{-1}$, so that the associated stroke lengths $u_{c}/\omega$ are much smaller than the characteristic longitudinal distance ≃2−4cm. Their ratio

(2.5) $$\varepsilon =\frac{u_{c}/\omega }{\lambda },$$

of order ε≃0.05 for spinal CSF flow, defines the small parameter employed in the following asymptotic description. As shown earlier (Hall 1974; Grotberg 1984), the solution at leading order is determined by a balance between the pressure gradient, the local acceleration and the viscous forces, with the convective acceleration introducing small corrections of relative magnitude ε. Although the leading-order motion is harmonic, the velocity corrections include a non-zero steady-streaming component.

The familiar periodic channel flow described previously is altered by gravitational forces when a solute of density $\rho_{s}\neq \rho$ is introduced in the channel. The extent of the resulting buoyancy-induced motion can be measured by the associated Richardson number

(2.6) $$Ri=\frac{\rho -\rho_{s}}{\rho }\frac{g\lambda }{u_{c}^{2}},$$

which compares the order of magnitude of the effective gravitational acceleration $g(\rho -\left.\rho_{s}\right)/\rho$ with that of the convective acceleration $v^{*}\cdot \nabla^{*}v^{*}\sim u_{c}^{2}/\lambda$. Our analysis addresses the limit Ri~1, which is relevant for drug dispersion in ITDD procedures, as can be seen by evaluating (2.6) with λ≃2cm and $u_{c}\sim 1\;cm{\;s}^{-1}$ for density differences in the range $10^{-3}≲\left|\rho -\rho_{s}\right|/\rho ≲10^{-2}$.

Also motivated by ITDD applications, we consider solutes with diffusivities κ much smaller than the kinematic viscosity, that always being the case of diffusion in liquid phase. As v/κ~1000 and ε≃0.05 in ITDD applications, the following analysis of solute dispersion will specifically address the distinguished limit $\kappa /\nu \sim \varepsilon^{2}$, with solute diffusion correspondingly characterised by the reduced Schmidt number

(2.7) $$\sigma =\varepsilon^{2}\frac{\nu }{\kappa },$$

assumed to be of order unity.

### 2.3. Non-dimensional formulation

We address the motion that follows from the deposition of the solute inside an intermediate cell along the channel. The problem is non-dimensionalised using the scales identified previously to give the dimensionless variables

(2.8a–f) $$t=\omega t^{*},\;x=\frac{x^{*}}{\lambda },\;y=\frac{y^{*}}{h_{o}},\;u=\frac{u^{*}}{u_{c}},\;v=\frac{v^{*}}{v_{c}},\;p=\frac{p^{*}}{\Delta p}$$

and associated conservation equations

(2.9) $$\frac{\partial u}{\partial x}+\frac{\partial v}{\partial y}=0,$$

(2.10) $$\frac{\partial u}{\partial t}+\varepsilon v\cdot \nabla u=-\frac{\partial p}{\partial x}+\frac{1}{\alpha^{2}}\frac{\partial^{2}u}{\partial y^{2}}-\varepsilon \text{Ri}c,$$

(2.11) $$\frac{\partial p}{\partial y}=0,$$

(2.12) $$\frac{\partial c}{\partial t}+\varepsilon v\cdot \nabla c=\frac{\varepsilon^{2}}{\alpha^{2}\sigma }\frac{\partial^{2}c}{\partial y^{2}},$$

where v=(u,v) and ∇=(∂/∂x,∂/∂y). In writing (2.9)–(2.12) from (2.1)–(2.3) we have used the slender-flow approximation resulting from the limit $h_{o}\ll \lambda$. Thus, the terms representing longitudinal diffusion of momentum and mass have been neglected in (2.10) and (2.12), because they are a factor ${\left(h_{o}/\lambda \right)}^{2}$ smaller than those associated with transverse diffusion. At the same level of approximation, the transverse component of the momentum equation takes the reduced form (2.11). The velocity and concentration must satisfy the boundary conditions

(2.13) $$u=v=\frac{\partial c}{\partial y}=0\;\text{ at }\left\{\begin{array}{l} y=0\\ y=H=1+\beta cos\;(2\pi x) \end{array},\right.$$

corresponding to non-permeable no-slip surfaces, whereas the reduced pressure p(x,t), independent of y, is identically zero at x=0 and takes the value p=ncos t at the lower end x=n.

In the absence of buoyancy (i.e. for Ri=0), the solution for the velocity is periodic in time, including a steady component of order ε, and also periodic in space, so that the velocity distribution found in each cell is identical. On the other hand, for Ri≠0 the motion is coupled to the solute transport, albeit weakly, with the result that the velocity necessarily evolves in time following the dispersion of the solute, which is driven partly by the steady streaming motion, with characteristic velocities $\varepsilon u_{c}$. It can be anticipated that the characteristic time for the slow evolution is that associated with the dispersion of the solute inside the deposition cell $\lambda /\left(\varepsilon u_{c}\right)=\varepsilon^{-2}\omega^{-1}$, much larger than the characteristic oscillation time $\omega^{-1}$. These considerations suggest the introduction of a second time variable $\tau =\varepsilon^{2}t$ for describing the slow evolution, additional to the fast time-scale variable t describing the oscillatory motion. In the two-time-scale formalism, the time derivatives in (2.10) and (2.12) are replaced with $\partial /\partial t+\varepsilon^{2}\partial /\partial \tau$ and the different variables are expressed in terms of the power expansions

(2.14) $$u=u_{0}(x,y,t,\tau )+\varepsilon u_{1}(x,y,t,\tau )+\cdots ,$$

(2.15) $$v=v_{0}(x,y,t,\tau )+\varepsilon v_{1}(x,y,t,\tau )+\cdots ,$$

(2.16) $$p=p_{0}(x,t,\tau )+\varepsilon p_{1}(x,t,\tau )+\cdots ,$$

(2.17) $$c=c_{0}(x,y,t,\tau )+\varepsilon c_{1}(x,y,t,\tau )+\cdots ,$$

with all functions assumed to be 2π periodic in the fast time scale t. The asymptotic procedure leads to a hierarchy of problems that can be solved sequentially, as shown in the following.

## 3. Velocity description

We begin by describing the velocity field in the asymptotic limit ε≪1, following the procedure used in previous steady-streaming investigations of slender flows (Larrieu *et al*. 2009; Guibert *et al*. 2010; Sánchez *et al*. 2018). The solution at leading order and also the first-order corrections associated with convective acceleration are similar to those found earlier in three-dimensional wavy-walled channels (Guibert *et al*. 2010) and annular canals (Sánchez *et al*. 2018). These previous analyses did not address, however, effects of buoyancy forces, which are investigated here for order-unity values of the Richardson number Ri, leading to a velocity correction that will be seen to be expressible in terms of integrals of the solute concentration.

### 3.1. Leading-order oscillatory flow

Convective acceleration and buoyancy are negligible at leading order, so that the velocity $v_{0}=\left(u_{0},v_{0}\right)$ satisfies a linear problem that can be solved in terms of the reduced variables

(3.1 a–c) $$u_{0}=Re\;\left({ie}^{it}U\right),\;v_{0}=Re\;\left({ie}^{it}V\right),\;p_{0}=Re\;\left(e^{it}P\right),$$

where the complex functions U(x,y),V(x,y), and P(x) satisfy

(3.2a,b) $$\frac{\partial U}{\partial x}+\frac{\partial V}{\partial y}=0\;\text{ and }\;-U=-\frac{dP}{\;dx}+\frac{i}{\alpha^{2}}\frac{\partial^{2}U}{\partial y^{2}}.$$

The second equation above can be integrated with boundary conditions U=0 at y=0,H to give

(3.3) $$U=\frac{dP}{\;dx}G(x,y),$$

where

(3.4a,b) $$G=1-\frac{cosh\;[\Lambda (2y/H-1)]}{cosh\;\Lambda }\text{ and }\Lambda =\frac{\alpha }{2}\frac{1+i}{\sqrt{2}}H(x).$$

The result can be used to integrate the first equation in (3.2a,b) subject to V=0 at y=0, yielding

(3.5) $$V=-\frac{\partial }{\partial x}\left(\int_{0}^{y}U\;d\overset{ˆ}{y}\right)=-\frac{\partial }{\partial x}\left(\frac{dP}{\;dx}\int_{0}^{y}G\;d\overset{ˆ}{y}\right),$$

where

(3.6) $$\int_{0}^{y}G\;d\overset{ˆ}{y}=y-\frac{H}{2\Lambda }\left\{\frac{sinh\;[\Lambda (2y/H-1)]+sinh\;\Lambda }{cosh\;\Lambda }\right\},$$

with $\overset{ˆ}{y}$ representing a dummy integration variable. Note that both velocity components U and V are spatially periodic in x through the function H=1+βcos (2πx). The determination of the longitudinal pressure gradient dP/dx that completes the solution begins by using the condition V=0 at y=H in the first equation of (3.5) to give

(3.7) $$\frac{d}{dx}\left(\int_{0}^{H}U\;dy\right)=0,$$

indicating that the reduced flow rate

(3.8) $$Q=\int_{0}^{H}U\;dy=\frac{dP}{\;dx}\int_{0}^{H}G\;dy$$

is constant. Further progress requires use of the conditions P(0)=P(n)−n=0, consistent with the boundary values p(0,t)=0 and p(n,t)=ncos t stated below (2.13). Using (3.6) to evaluate the integral $\int_{0}^{H}G\;dy$ leads to the equation

(3.9) $$Q=\frac{dP}{\;dx}H\left(1-\Lambda^{-1}tanh\;\Lambda \right),$$

which can be integrated subject to P(0)=0 to yield the pressure distribution

(3.10) $$P=Q\int_{0}^{x}\frac{d\overset{ˆ}{x}}{H\left(1-\Lambda^{-1}tanh\;\Lambda \right)}.$$

Using now the condition P(n)=n provides

(3.11) $$Q=n{\left[\int_{0}^{n}\frac{dx}{H\left(1-\Lambda^{-1}tanh\;\Lambda \right)}\right]}^{-1}.$$

Owing to the spatial periodicity of H and Λ, it follows that

(3.12) $$\int_{0}^{n}\frac{dx}{H\left(1-\Lambda^{-1}tanh\;\Lambda \right)}=n\int_{0}^{1}\frac{dx}{H\left(1-\Lambda^{-1}tanh\;\Lambda \right)},$$

thereby finally yielding

(3.13) $$Q={\left[\int_{0}^{1}\frac{dx}{H\left(1-\Lambda^{-1}tanh\;\Lambda \right)}\right]}^{-1}$$

and, from (3.9),

(3.14) $$\frac{dP}{\;dx}={\left[H\left(1-\Lambda^{-1}tanh\;\Lambda \right)\int_{0}^{1}\frac{dx}{H\left(1-\Lambda^{-1}tanh\;\Lambda \right)}\right]}^{-1},$$

independent of n. It is worth pointing out that, because at this order the velocity is harmonic, the associated time-averaged values $\left\langle u_{0}\right\rangle$ and $\left\langle v_{0}\right\rangle$ with $\langle \cdot \rangle =(1/2\pi )\int_{t}^{t+2\pi }dt$ are identically zero, so that the steady bulk motion of the fluid occurs through the velocity corrections at the following order.

### 3.2. First-order corrections

Collecting terms of order ε in (2.9) and (2.10) yields

(3.15) $$\frac{\partial u_{1}}{\partial x}+\frac{\partial v_{1}}{\partial y}=0,$$

(3.16) $$\frac{\partial u_{1}}{\partial t}+\frac{\partial }{\partial x}\left(u_{0}^{2}\right)+\frac{\partial }{\partial y}\left(u_{0}v_{0}\right)=-\frac{\partial p_{1}}{\partial x}+\frac{1}{\alpha^{2}}\frac{\partial^{2}u_{1}}{\partial y^{2}}-Ric_{0},$$

to be integrated with boundary conditions $u_{1}=v_{1}=0$ at y=0,H and $p_{1}=0$ at x=0,n. There is interest in computing the corresponding time-averaged velocity correction $\left\langle v_{1}\right\rangle =\left(\left\langle u_{1}\right\rangle ,\left\langle v_{1}\right\rangle \right)$. Taking the time average of (3.15) and (3.16) provides

(3.17 a,b) $$\frac{\partial \left\langle u_{1}\right\rangle }{\partial x}+\frac{\partial \left\langle v_{1}\right\rangle }{\partial y}=0\;\text{ and }\;F(x,y)=-\frac{\partial \left\langle p_{1}\right\rangle }{\partial x}+\frac{1}{\alpha^{2}}\frac{\partial^{2}\left\langle u_{1}\right\rangle }{\partial y^{2}}-Ric_{0},$$

where the known function $F=\partial \left\langle u_{0}^{2}\right\rangle /\partial x+\partial \left\langle u_{0}v_{0}\right\rangle /\partial y$ can be expressed in terms of the complex velocities U and V defined above in the form

(3.18) $$F=\frac{1}{2}Re\;\left[\frac{\partial }{\partial x}(U\overset{‾}{U})+\frac{\partial }{\partial y}(V\overset{‾}{U})\right],$$

a result following from the identity $\left\langle Re\;\left({ie}^{it}f_{1}\right)Re\;\left({ie}^{it}f_{2}\right)\right\rangle =Re\;\left(f_{1}{\overset{‾}{f}}_{2}\right)/2$, which applies to any generic time-independent complex functions $f_{1}$ and $f_{2}$, with the bar denoting complex conjugates. In writing (3.17a,b), we have anticipated that, at leading order, the solute concentration is independent of the fast time scale t, as follows from (2.12) when ε≪1, so that its time-averaged value $\left\langle c_{0}\right\rangle$ reduces simply to $\left\langle c_{0}\right\rangle =c_{0}$. Also of interest is that, because of the symmetry of H(x), the periodic function F defined in (3.18) is antisymmetric with respect to x=1/2, so that F(x,y)=−F(1−x,y).

As can be concluded from (3.16), the velocity corrections arise partly owing to the convective acceleration and partly owing to the buoyancy force. In computing the corresponding time average, it is convenient to compute both contributions separately by introducing $\left\langle v_{1}\right\rangle =v_{SS}+v_{B}$ and $\left\langle p_{1}\right\rangle =p_{SS}+p_{B}$, where $v_{SS}(x,y)=\left(u_{SS},v_{SS}\right)$ and $p_{SS}(x)$ describe the familiar steady-streaming associated with the nonlinear convective terms, which is independent of time and periodic in x, and $v_{B}(x,y,\tau )=\left(u_{B},v_{B}\right)$ and $p_{B}(x,\tau )$ describe the buoyancy-induced corrections, which evolve in the long time scale τ as the solute spreads in the channel.

### 3.3. Steady streaming

The solution procedure needed to compute the velocity corrections parallels that followed earlier at leading order. Thus, the longitudinal component of the steady-streaming velocity

(3.19) $$\frac{u_{SS}}{\alpha^{2}}=-\frac{dpp_{SS}}{\;dx}\frac{1}{2}(H-y)y+y\int_{0}^{y}F\;d\overset{ˆ}{y}-\int_{0}^{y}F\overset{ˆ}{y}\;d\overset{ˆ}{y}-y\int_{0}^{H}F\left(1-\frac{y}{H}\right)dy$$

is determined by integrating the momentum equation (3.16) written for Ri=0 subject to the boundary conditions $u_{SS}=0$ at y=0,H. The result can be substituted into (3.15) to give

(3.20) $$\frac{v_{SS}}{\alpha^{2}}=\frac{\partial }{\partial x}[\frac{dp_{SS}}{\;dx}\frac{y^{2}}{2}\left(\frac{H}{2}-\frac{y}{3}\right)+\frac{y^{2}}{2}\int_{H}^{y}F\left(1-\frac{\hat{y}}{H}\right)d\hat{y}+y\left(1-\frac{y}{2H}\right)\int_{y}^{0}F\hat{y}\;d\hat{y}-\frac{1}{2}\int_{y}^{0}F{\hat{y}}^{2}\;d\hat{y}]$$

upon integrating with $v_{SS}=0$ at y=0. To determine the unknown pressure gradient $dp_{SS}/dx$ we begin by using $v_{SS}=0$ at y=H in the above equation to give

(3.21) $$\frac{d}{dx}\left(\int_{0}^{H}\frac{u_{SS}}{\alpha^{2}}\;dy\right)=\frac{d}{dx}\left(\frac{dp_{SS}}{\;dx}\frac{H^{3}}{12}+\frac{1}{2}\int_{0}^{H}Fy(H-y)dy\right)=0,$$

which indicates that the flow rate

(3.22) $$Q_{SS}=\int_{0}^{H}u_{SS}\;dy=\alpha^{2}\left[\frac{dp_{SS}}{\;dx}\frac{H^{3}}{12}+\frac{1}{2}\int_{0}^{H}Fy(H-y)dy\right]$$

is constant. Its value can be determined by integrating a second time (3.22) subject to $p_{SS}(n)=p_{SS}(0)=0$ to yield

(3.23) $$Q_{SS}=\frac{\alpha^{2}\int_{0}^{1}H^{-3}\left[\int_{0}^{H}Fy(H-y)dy\right]dx}{2\int_{0}^{1}H^{-3}\;dx},$$

when account is taken of the spatial periodicity of H and F. Since H(x) is symmetric about x=1/2 while F(x,y) is antisymmetric, the double integral in the numerator of the above equation is identically zero, so that

(3.24) $$Q_{SS}=\int_{0}^{H}u_{SS}\;dy=0,$$

in agreement with previous findings regarding steady streaming in tubes (Hall 1974) and channels (Lo Jacono *et al*. 2005; Guibert *et al*. 2010). Using the condition $Q_{SS}=0$ in (3.22) finally yields

(3.25) $$\frac{dp_{SS}}{\;dx}=-6H^{-3}\int_{0}^{H}Fy(H-y)dy,$$

for the pressure gradient, thereby completing the solution.

### 3.4. Buoyancy-induced velocity

The corresponding solution for the buoyancy-induced velocity can be obtained by simply replacing F(x,y) with $Ri\;c_{0}(x,y,\tau )$ in (3.19) and (3.20), yielding

(3.26) $$\frac{u_{B}}{\alpha^{2}Ri}=-\frac{1}{Ri}\frac{\partial p_{B}}{\partial x}\frac{1}{2}(H-y)y+y\int_{0}^{y}c_{0}\;d\overset{ˆ}{y}-\int_{0}^{y}c_{0}\overset{ˆ}{y}\;d\overset{ˆ}{y}-y\int_{0}^{H}c_{0}\left(1-\frac{y}{H}\right)dy$$

and

(3.27) $$\frac{v_{B}}{\alpha^{2}Ri}=\frac{\partial }{\partial x}\left[\frac{1}{Ri}\frac{\partial p_{B}}{\partial x}\frac{y^{2}}{2}\left(\frac{H}{2}-\frac{y}{3}\right)+\frac{y^{2}}{2}\int_{y}^{H}c_{0}\left(1-\frac{\overset{ˆ}{y}}{H}\right)d\overset{ˆ}{y}\right.\left.+y\left(1-\frac{y}{2H}\right)\int_{0}^{y}c_{0}\overset{ˆ}{y}\;d\overset{ˆ}{y}-\frac{1}{2}\int_{0}^{y}c_{0}{\overset{ˆ}{y}}^{2}\;d\overset{ˆ}{y}\right].$$

Using the condition $v_{B}=0$ at y=H in the above equation and integrating once gives

(3.28) $$\frac{Q_{B}}{\alpha^{2}Ri}=\frac{1}{Ri}\frac{\partial p_{B}}{\partial x}\frac{H^{3}}{12}+\frac{1}{2}\int_{0}^{H}c_{0}y(H-y)dy$$

for the buoyancy-induced flow rate $Q_{B}=\int_{0}^{H}u_{B}$ dy. Integrating a second time with $p_{B}=0$ at x=0,n to give

(3.29) $$Q_{B}(\tau )=\frac{\alpha^{2}Ri\int_{0}^{n}H^{-3}\left[\int_{0}^{H}c_{0}y(H-y)dy\right]dx}{2n\int_{0}^{1}H^{-3}\;dx},$$

finally determines the pressure gradient

(3.30) $$\frac{1}{Ri}\frac{\partial p_{B}}{\partial x}=\frac{6}{H^{3}}\left\{\frac{\int_{0}^{n}H^{-3}\left[\int_{0}^{H}c_{0}y(H-y)dy\right]dx}{n\int_{0}^{1}H^{-3}\;dx}-\int_{0}^{H}c_{0}y(H-y)dy\right\},$$

which can be used in (3.26) and (3.27) to complete the determination of the buoyancy-induced velocity. Note that, because $c_{0}$ is not spatially periodic, the solution carries a dependence on the channel length n through the pressure gradient $\partial p_{B}/\partial x$.

## 4. Solute dispersion

The flow velocity is coupled with the solute concentration c through the dependence on $c_{0}$ present in $v_{B}=\left(u_{B},v_{B}\right)$. The computation of $c_{0}$ involves substitution of the expansion $c=c_{0}+\varepsilon c_{1}+\varepsilon^{2}c_{2}+\cdots$ into (2.12) with the time derivative replaced by the two-time-scale expression $\partial c/\partial t+\varepsilon^{2}\partial c/\partial \tau$. At leading order we find the result $\partial c_{0}/\partial t=0$, anticipated earlier when writing (3.17a,b). Collecting terms of order ε yields

(4.1) $$\frac{\partial c_{1}}{\partial t}+v_{0}\cdot \nabla c_{0}=0,$$

which can be integrated to provide the concentration correction

(4.2) $$c_{1}=\left\langle c_{1}\right\rangle (x,y,\tau )-\int \;v_{0}\;dt\cdot \nabla c_{0},$$

where $\int v_{0}\;dt=Re\;\left[e^{it}(U,V)\right]$, as follows from (3.1a-c). It is worth noting that, because the solute diffusivity takes small values of order $\kappa /\nu \sim \varepsilon^{2}$, effects of diffusion are absent at the first two orders in the asymptotic analysis. These effects are present in the equation that arises at the following order,

(4.3) $$\frac{\partial c_{2}}{\partial t}+\frac{\partial c_{0}}{\partial \tau }+v_{0}\cdot \nabla c_{1}+v_{1}\cdot \nabla c_{0}=\frac{1}{\alpha^{2}\sigma }\frac{\partial^{2}c_{0}}{\partial y^{2}},$$

which can be time-averaged to give

(4.4) $$\frac{\partial c_{0}}{\partial \tau }+\left(\left\langle \int \;v_{0}\;dt\cdot \nabla v_{0}\right\rangle +\left\langle v_{1}\right\rangle \right)\cdot \nabla c_{0}=\frac{1}{\alpha^{2}\sigma }\frac{\partial^{2}c_{0}}{\partial y^{2}}.$$

In deriving the second term in (4.4) from the third term in (4.3) use has been made of (4.2). Since $\left\langle c_{1}\right\rangle$ is independent of t and $\left\langle v_{0}\right\rangle =0$, the contribution of the former to the resulting time average $\left\langle \left(v_{0}\cdot \nabla \left\langle c_{1}\right\rangle \right)\right\rangle =\left\langle v_{0}\right\rangle \cdot \nabla \left\langle c_{1}\right\rangle$ is identically zero. The leading-order solute concentration $c_{0}$ is also independent of t, so that the contribution arising from the second term in (4.2) can be written in the form

(4.5) $$-\left\langle v_{0}\cdot \nabla \left(\int \;v_{0}\;dt\cdot \nabla c_{0}\right)\right\rangle =-\left\langle v_{0}\cdot \int \;\nabla u_{0}\;dt\right\rangle \frac{\partial c}{\partial x}-\left\langle v_{0}\cdot \int \;\nabla v_{0}\;dt\right\rangle \frac{\partial c}{\partial y}\;-\left\langle u_{0}\int \;u_{0}\;dt\right\rangle \frac{\partial^{2}c}{\partial x^{2}}-\left\langle v_{0}\int \;u_{0}\;dt+u_{0}\int \;v_{0}\;dt\right\rangle \frac{\partial^{2}c}{\partial x\partial y}-\left\langle v_{0}\int \;v_{0}\;dt\right\rangle \frac{\partial^{2}c}{\partial y^{2}}.$$

With the time averages of any two harmonic functions A and B satisfying $\left\langle A\left(\int B\;dt\right)\right\rangle =-\left\langle \left(\int A\;dt\right)B\right\rangle$ and $\left\langle A\left(\int A\;dt\right)\right\rangle =\left\langle B\left(\int B\;dt\right)\right\rangle =0$, it follows that the terms in the second line of the above equation are identically zero, whereas the remaining two terms on the right-hand side can be cast in the compact form shown in (4.4).

As seen in (4.4), convective transport in the long time scale relies on the time-averaged Lagrangian velocity, given by the sum of the time-averaged Eulerian velocity $\left\langle v_{1}\right\rangle =v_{SS}+v_{B}$ and the Stokes drift $v_{SD}=\left(u_{SD},v_{SD}\right)=\left\langle \int v_{0}\;dt\cdot \nabla v_{0}\right\rangle$ (see, e.g., Larrieu *et al*. 2009 for a discussion on Lagrangian transport in a similar wall-bounded flow). The latter contribution can be evaluated in terms of the complex functions U and V with use of the expressions

(4.6a,b) $$u_{SD}=\frac{1}{2}Im\;\left[\frac{\partial }{\partial x}(U\overset{‾}{U})+\frac{\partial }{\partial y}(V\overset{‾}{U})\right]\;\text{ and }\;v_{SD}=\frac{1}{2}Im\;\left[\frac{\partial }{\partial x}(U\overset{‾}{V})+\frac{\partial }{\partial y}(V\overset{‾}{V})\right],$$

which follow from the identity $\left\langle Re\;\left(e^{it}f_{1}\right)Re\;\left(i{\;}^{it}f_{2}\right)\right\rangle =Im\;\left(f_{1}{\overset{‾}{f}}_{2}\right)/2$. The function $u_{SD}$, which is related to the function F defined earlier in (3.18), is identically zero at x=0,1/2,1,3/2,…, so that the associated constant volumetric flow rate is simply

(4.7) $$Q_{SD}=\int_{0}^{H}u_{SD}\;dy=0.$$

As our asymptotic description is limited to the leading-order term in the asymptotic expansion (2.17) for the solute concentration, to summarise the results of the asymptotic analysis one may replace $c_{0}$ with c when rewriting the final transport (4.4) in the form

(4.8) $$\frac{\partial c}{\partial \tau }+\left(u_{SD}+u_{SS}+u_{B}\right)\frac{\partial c}{\partial x}+\left(v_{SD}+v_{SS}+v_{B}\right)\frac{\partial c}{\partial y}=\frac{1}{\alpha^{2}\sigma }\frac{\partial^{2}c}{\partial y^{2}}.$$

The description of the solute dispersion following its deposition in the channel reduces to the integration of the above equation with initial condition $c=c_{i}(x,y)$ at τ=0 and boundary conditions ∂c/∂y=0 at y=0,H. In the integration, the time-independent Stokes-drift and steady-streaming velocities are computed with use of (4.6a,b) and of (3.19), (3.20) and (3.25), respectively, whereas the time-varying buoyancy-induced velocity is evaluated in terms of the solute concentration through the integral expressions (3.26), (3.27) and (3.30) with $c_{0}=c$. Observation of (4.8) reveals that gravitational forces modify the character of the solution in a non-trivial way. Owing to the dependence of $u_{B}$ and $v_{B}$ on the concentration distribution c, the time-averaged transport equation that governs the dispersion of the solute, which for Ri=0 reduces to a linear partial-differential equation with time-independent coefficients, turns into a complicated nonlinear integro-differential equation in the presence of buoyancy.

It is worth noting that, whereas the volumetric flow rates $Q_{SS}=\int_{0}^{H}u_{SS}\;dy$ and $Q_{SD}=\int_{0}^{H}u_{SD}\;dy$ associated with steady streaming and Stokes drift are identically zero, as discussed previously, that induced by buoyancy is in general non-zero, its value $Q_{B}=\int_{0}^{H}u_{B}\;dy\;$ evolving in time according to (3.29). Note that writing (4.8) in conservative form and integrating across the channel with use of ∂c/∂y=0 and $v_{SD}=v_{SS}=v_{B}=0$ at y=0,H yields the relation

(4.9) $$\frac{\partial C}{\partial \tau }+\frac{\partial \phi }{\partial x}=0$$

between the amount of solute per unit channel length $C(x,\tau )=\int_{0}^{H}c\;dy$ and the solute flux $\phi (x,\tau )=\int_{0}^{H}\left(u_{SD}+u_{SS}+u_{B}\right)c\;dy$, whereas a second integral between x=0 and x=1 gives

(4.10) $$\frac{\partial }{\partial \tau }\left(\int_{0}^{1}C\;dx\right)+\phi (1,\tau )-\phi (0,\tau )=0,$$

which naturally reduces to the expected conservation law

(4.11) $$\int_{0}^{1}\left(\int_{0}^{H}c\;dy\right)dx=\int_{0}^{1}\left(\int_{0}^{H}c_{i}\;dy\right)dx,$$

when the solute flux at the two ends is zero.

An important aspect of the reduced description developed above is that the nonlinear integro-differential equation (4.8) targets directly the evolution of the flow in the long time scale τ~1, relevant for solute dispersion over distances of order λ (i.e. dimensionless distances x of order unity), thereby circumventing the need to describe the small concentration fluctuations occurring in the short time scale $t=\varepsilon^{-2}\tau$. As a consequence, the model predictions involve computational times that can be expected to be a factor $\varepsilon^{2}$ smaller than those required in DNS, because to describe solute dispersion the DNS must track the flow over a large number of cycles $\sim \varepsilon^{-2}$, larger for smaller ε.

## 5. Selected numerical results

The reduced flow description is to be utilised to investigate the influence of buoyancy on solute dispersion. To facilitate the computation, the conservation (4.8) was written in terms of the normalised coordinate η=y/H(x), so that the integration domain becomes 0<x<n and 0<η<1. The numerical scheme utilises a fourth-order compact centred finite-difference approximation for the spatial discretisations of the viscous terms and a second-order upwind scheme for the nonlinear terms. A third-order TVD Runge–Kutta scheme is used for the time marching, whereas the integral expressions (3.26), (3.27) and (3.30) are evaluated with a simple trapezoidal rule.

The accuracy of the model predictions, derived in the asymptotic limit ε≪1 for a slender channel with $h_{o}/\lambda \ll 1$, is tested through comparisons with two-dimensional, unsteady simulations of the fluid motion and solute dispersion for small but finite values of $h_{o}/\lambda$ and ε. The DNS, involving the complete (2.1)–(2.3) written in dimensionless form, span thousands of oscillation cycles, as needed to generate significant dispersion of the solute. The numerical integration was performed with the finite-volume solver Ansys Fluent (release 20.2), assuring second-order accuracy in time and in space. Computations employing upwind and central-differencing schemes for the convective terms were found to yield virtually indistinguishable results, with the former discretisation used in generating the figures shown in the following. A coupled algorithm was used for the pressure–velocity coupling. In addition to the boundary conditions used in integrating the slender flow (2.9)–(2.12), additional conditions of developed flow ∂u/∂x=∂c/∂x=0 at the upper and lower open boundaries are incorporated in integrating (2.1)–(2.3). The computations presented in the following correspond to a canal of total length n=3 and aspect ratio $h_{o}/\lambda =1/20$ with the imposed pressure difference yielding a dimensionless stroke length ε=0.02. The time-periodic DNS results were averaged in time to determine the mean Eulerian velocity $\langle v\rangle =(1/2\pi )\int_{t}^{t+2\pi }v\;dt$, of order ε, to be compared with the steady-streaming velocity $v_{SS}$, as explained later. In addition, tracer particles are used to compute the Lagrangian velocity $v_{L}$ by following their displacement over a cycle, i.e. if the particle located at (x,y) at time t moves to occupy the new location (x+δx,y+δy) at time t+2π, then the Lagrangian velocity at (x,y) and time t is defined as $v_{L}=(\delta x,\delta y)/(2\pi )$.

### 5.1. Buoyancy-free flow

**Figure 2.**
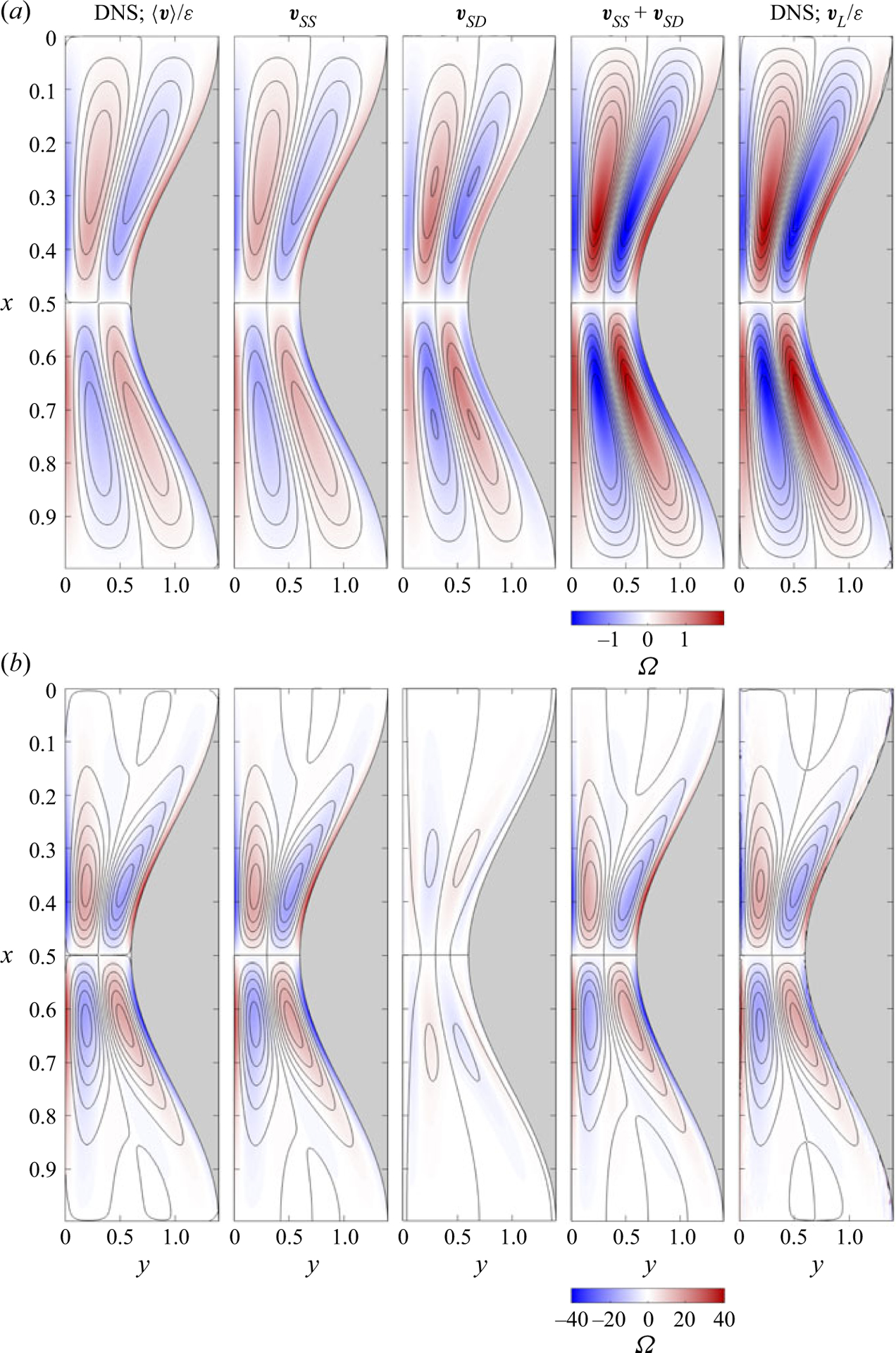
Streamlines and colour contours of vorticity corresponding to the steady-streaming velocity $v_{SS}$, Stokes-drift velocity $v_{SD}$ and steady mean Lagrangian velocity $v_{SS}+v_{SD}$ in a canal with β=0.4 for (a) α=4 and (b) α=16. Results of DNS of a non-buoyant flow (i.e. Ri=0) with $h_{o}/\lambda =1/20$ and ε=0.02 are also shown, including the rescaled time-averaged Eulerian velocity field ⟨v⟩/ε (first column) and the rescaled Lagrangian velocity $v_{L}/\varepsilon$ (fifth column), the latter determined by following tracer particles, as explained in the text. To facilitate the comparisons, fixed constant streamline spacings δψ=0.003 and δψ=0.03 are used for the upper and lower plots, respectively.

As mentioned previously, in the absence of buoyancy, the flow induced by the imposed pressure gradient is periodic in time and space. The steady Lagrangian motion for ε≪1 is given in this case by the sum of the steady-streaming velocity $v_{SS}$ and the Stokes-drift velocity $v_{SD}$. These two contributions as well as their sum are shown in figure 2 for β=0.4 and two different values of α. As the flow in each cell is identical, it suffices to show the solution for 0≤x≤1, symmetric with respect to the centre line x=0.5. For each value of α, streamlines are plotted using a fixed increment δψ of the streamfunction ψ, defined in the usual way (e.g. $\partial \psi /\partial y=u_{SS}$ and $\partial \psi /\partial x=-v_{SS}$ for steady streaming) with ψ=0 along the wall, so that the interline spacing provides a measure of the local velocity. To further quantify the motion, colour contours are used to represent the associated vorticity $\Omega ={\left(h_{o}/\lambda \right)}^{2}\partial v/\partial x-\partial u/\partial y$, which reduces to Ω=−∂u/∂y in the slender flow approximation.

The spatially periodic, time-independent, steady-streaming velocity computed with (3.19) and (3.20) supplemented with (3.25) is shown in the second column of figure 2. The results are qualitatively similar to those presented in Guibert *et al*. (2010). For α=4, the flow structure of each half cell exhibits two counter-rotating vortices, whereas for α=16 the flow develops an additional, much weaker vortex, located near the section with largest width. As expected, the vorticity, having peak values of order unity for α=4, increases with increasing flow frequency as a result of augmented wall production to reach peak values exceeding Ω=40 for α=16.

The steady-streaming results are compared with time-averaged velocity fields obtained in DNS with ε=0.02. In the comparison, the time-averaged DNS velocity is expressed in the rescaled form ⟨v⟩/ε~1, consistent with the scaling employed in defining $v_{SS}$. The two functions $v_{SS}$ and ⟨v⟩/ε are seen to be almost identical, thereby giving additional confidence in the mathematical development. For instance, the peak values of the stream function and vorticity corresponding to the time-averaged DNS velocity ⟨v⟩/ε are $\psi_{\text{PEAK}}=\pm (0.0115,0.1680)$ and $\Omega_{\text{PEAK}}=\pm (1.4465,40.786)$ for α=(4,16), whereas the corresponding values for the steady-streaming motion are $\psi_{\text{PEAK}}=\pm (0.0115,0.1699)$ and $\Omega_{\text{PEAK}}=\pm (1.4474,40.787)$. The small relative differences remain below about 1%, as is consistent with the order of the asymptotic development.

The third column in figure 2 displays the Stokes-drift velocity field evaluated with (4.6a,b). As it is clear from a quantitative comparison with the corresponding steady-streaming results, both bulk-flow velocities have comparable magnitude for α=4, whereas for α=16 the Stokes drift provides a much smaller relative contribution to the Lagrangian drift. The dominant role of steady streaming in flows at high Womersley numbers is found also away from the wall in oscillating flow over circular cylinders (Holtsmark *et al*. 1954; Raney, Corelli & Westervelt 1954), for example. The mean Lagrangian velocity $v_{SS}+v_{SD}$ corresponding to the asymptotic limit ε≪1 compares favourably with the velocity $v_{L}/\varepsilon$ obtained numerically by following tracer particles in the DNS computation for ε=0.02, shown in the last column of figure 2, although the relative errors are somewhat larger than those of the Eulerian velocity. For instance, the peak values of the stream function and vorticity corresponding to $v_{L}/\varepsilon$ are $\psi_{\text{PEAK}}=\pm (0.0235,0.1674)$ and $\Omega_{\text{PEAK}}=\pm (2.0454,45.9497)$ for α=(4,16), while the corresponding values for $v_{SS}+v_{SD}$ are $\psi_{\text{PEAK}}=\pm (0.0235,0.1491)$ and $\Omega_{\text{PEAK}}=\pm (1.9906,40.787).$

### 5.2. Buoyancy-free solute dispersion

**Figure 3.**
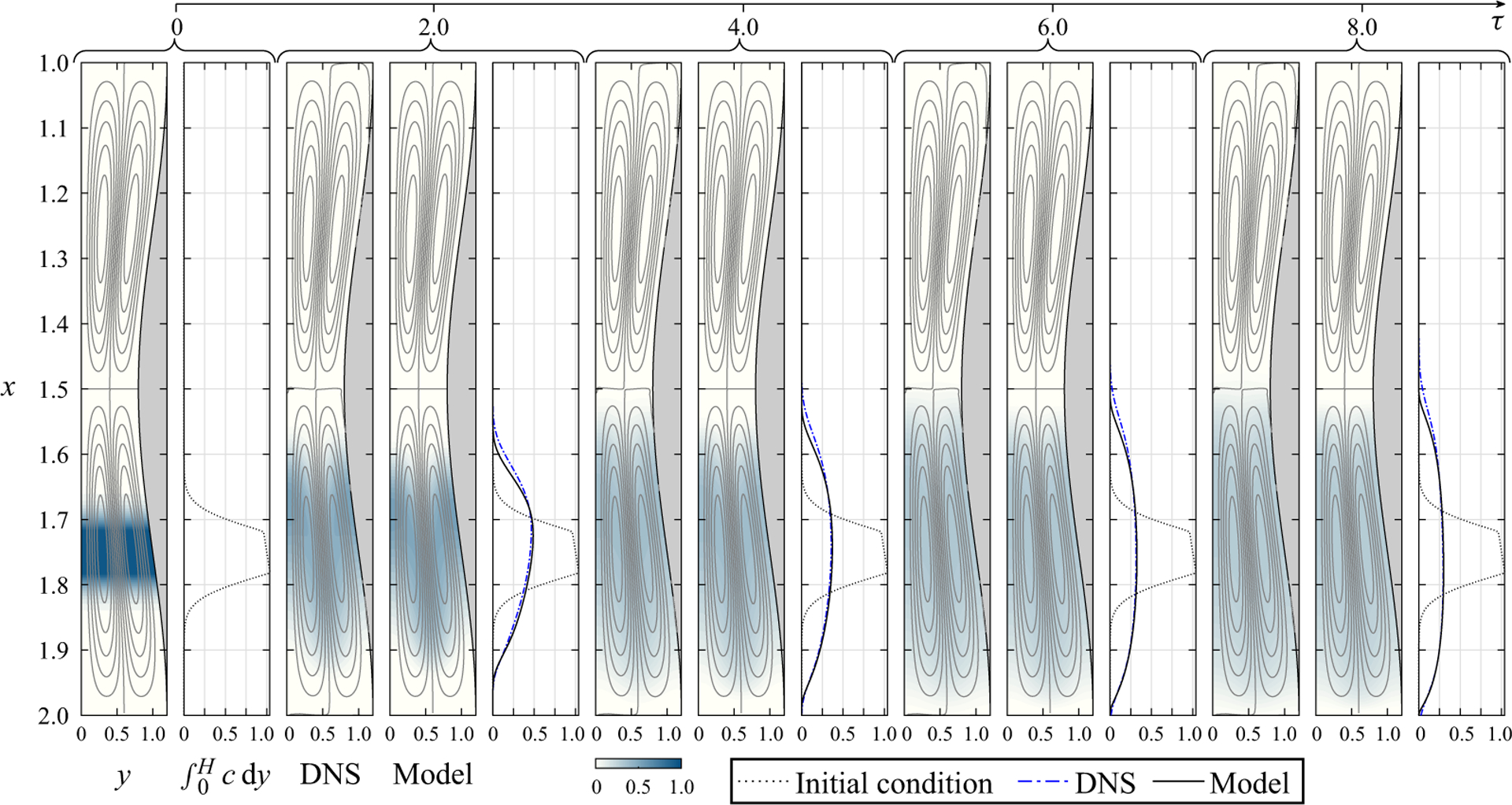
Snapshots of solute concentration for Ri=0,β=0.2,α=4 and σ=1 as obtained at five different instants of time from the reduced model and from DNS for $h_{o}/\lambda =1/20$ and ε=0.02. In addition to colour contours of local concentration, the figure shows distributions of solute per unit channel length $\int_{0}^{H}c\;dy$ for the model (solid curve) and for the DNS (dot-dashed curves), with the initial distribution $\int_{0}^{H}c_{i}\;dy$ shown as a dotted curve. For reference, the figure shows streamlines with constant spacing δψ=0.003 for the Lagrangian mean drift, which is characterised using the asymptotic prediction $v_{SS}+v_{SD}$ for the model results and the value of $v_{L}/\varepsilon$ determined numerically for the DNS results.

The reduced transport (4.8) resulting from the two-time-scale asymptotic analysis indicates that the solute relies on the Lagrangian drift for longitudinal dispersion. As a consequence, the existence of the closed recirculating vortices displayed in figure 2 implies that in oscillatory buoyancy-free channel flow a solute released in a given cell would be unable to reach their neighbouring cells, thereby precluding its progression along the canal. To illustrate this important feature of the flow, we show in figure 3 the temporal evolution of a bolus of solute with reduced Schmidt number σ=1 released at the initial instant of time in the central cell of a three-cell canal. The initial concentration is given by the truncated Gaussian distribution

(5.1) $$c_{i}=min\left\{1,\frac{3}{2}exp\;\left[-16{\left(\frac{x-x_{0}}{\delta }\right)}^{2}\right]\right\},$$

which represents a band of solute with characteristic width δ centred at $x_{o}$ having a saturated core flanked by thin layers across which the concentration decays to zero. Results obtained by integration of (4.8) for $x_{0}=1.75$ and δ=0.2 are compared in figure 3 at different instants of time in the interval 0≤τ≤8 with DNS computations. Note that, with $\tau =\varepsilon^{2}t$, for the value ε=0.02 used in the DNS, this interval of time corresponds to 0≤t≤20000 (i.e. about 20000/2π≃3200 oscillatory cycles).

As seen in figure 3 the model accurately reproduces the DNS results. To facilitate the quantitative comparisons, in addition to colour contours showing the solute concentration, the figure includes side plots for the amount of solute per unit channel length $C=\int_{0}^{H}c$ dy at different instants of time, with the initial distribution $C_{i}=H(x)c_{i}(x)$ included for reference as a dotted curve. The model predictions lie very close to the DNS results, in that the normalised value of the integrated departure $\left(\int_{0}^{n}\left|C_{DN}-C_{\text{MODEL}}\right|dx\right)/\left(\int_{0}^{n}C_{i}\;dx\right)$, which provides a metric for the accuracy of the model, remains below 0.003 over the entire range of times considered in the figure.

For the buoyancy-free conditions considered in figure 3, the steady Lagrangian motion is seen to stir the solute about the deposition location, uniformising its concentration within the recirculating cell. The effect of longitudinal diffusion, present in the DNS results, is found to be rather limited, in that, even at the latest instant of time considered, the presence of the solute in the adjacent cells is negligibly small. This tendency of the solute to remain trapped inside Lagrangian vortices has potential implications concerning the drug-dispersion rate in ITDD procedures. Although the Lagrangian flow in the spinal canal does not exhibit the spatial periodicity of the canonical configuration investigated here, closed recirculating vortices, associated with the changes in the eccentricity of the spinal cord along the canal, have been found to characterise the CSF bulk motion (Coenen *et al*. 2019). Typically, there are three main vortices, extending along the cervical, thoracic and lumbar regions. As ITDD injection occurs in the lumbar region, the buoyancy-free results in figure 3 seem to indicate that, when the density of the drug matches exactly the CSF density, the drug is bound to linger in the lumbar vortex near the injection site without reaching the thoracic region. This could be advantageous in applications involving pain medication, which is meant to be delivered to the spinal cord, but not in applications involving anticancer drugs targeting brain tumors, for example. As shown in the following, buoyancy-induced motion has the potential to drastically change the associated transport rate, in accordance with clinical observations (Mitchell *et al*. 1988; Povey *et al*. 1989; Richardson *et al*. 1996; Veering *et al*. 2001).

### 5.3. Slowly varying buoyancy-induced motion

**Figure 4.**
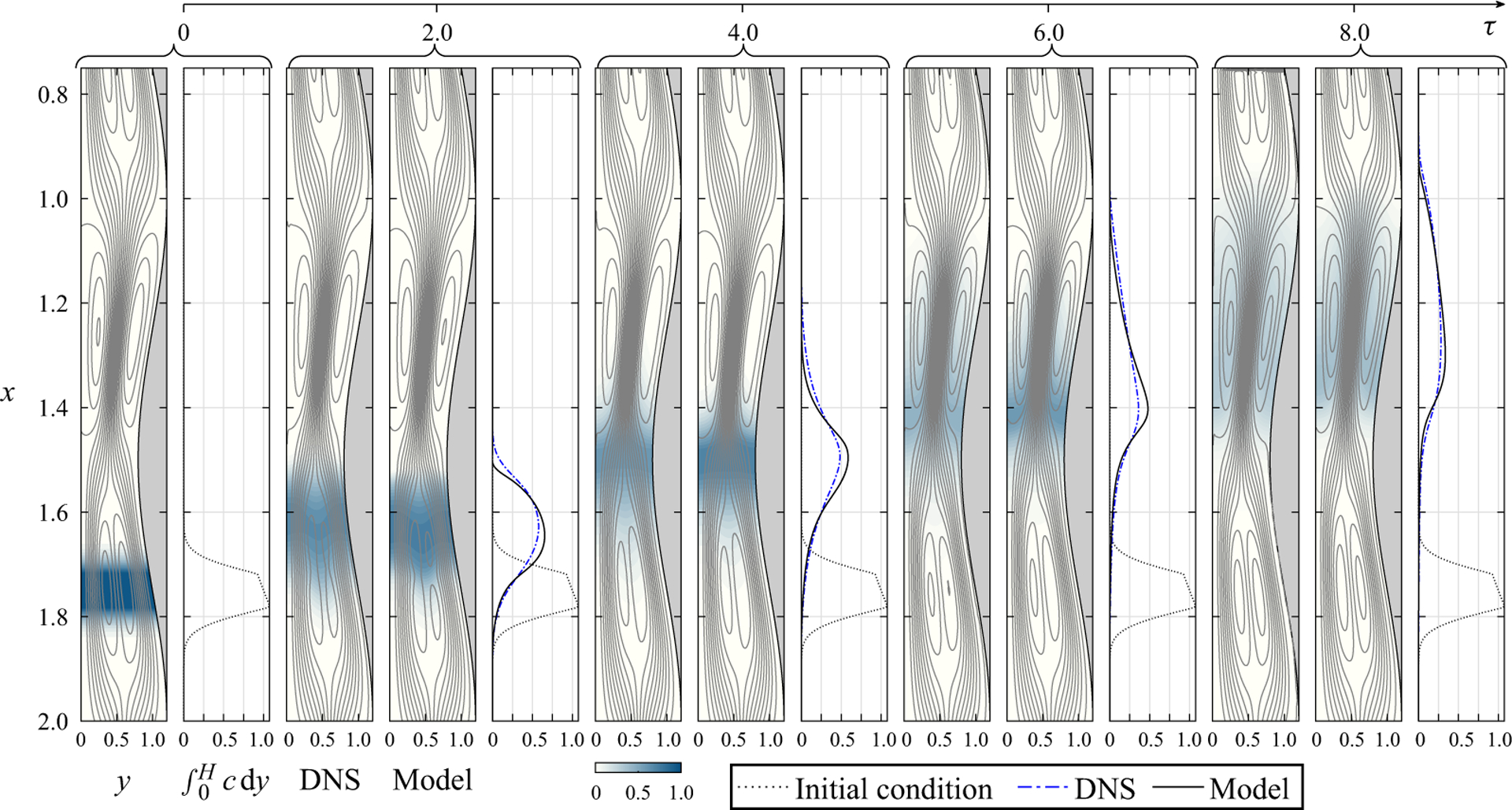
Snapshots of solute concentration for Ri=1,β=0.2,α=4 and σ=1 as obtained at five different instants of time from the reduced model and from DNS for $h_{o}/\lambda =1/20$ and ε=0.02. In addition to colour contours of local concentration, the figure shows distributions of solute per unit channel length $\int_{0}^{H}c\;dy$ for the model (solid curve) and for the DNS (dot-dashed curves), with the initial distribution $\int_{0}^{H}c_{i}\;dy$ shown as a dotted curve. The plots include streamlines with constant spacing δψ=0.003 for the varying Lagrangian mean drift, which is evaluated with use of $v_{SS}+v_{SD}+v_{B}$ (model results) and from the displacement of the tracer particles (DNS results).

As reasoned previously, buoyancy forces, acting on solutes with density $\rho_{s}\neq \rho$, alter the steady Lagrangian drift by adding an additional component that varies slowly in the long time scale τ following the solute dispersion, so that the flow and the solute transport are intimately coupled, as described by (4.8) supplemented with (3.26), (3.27) and (3.30). The corresponding behaviour is characterised in figure 4 for a light solute with Ri=1 spreading upwards. Note that, because of the problem symmetry, results corresponding to a heavy solute with Ri=−1 can be generated by simply reversing the direction of the gravity vector, i.e. by rotating the figure 180°.

**Figure 5.**
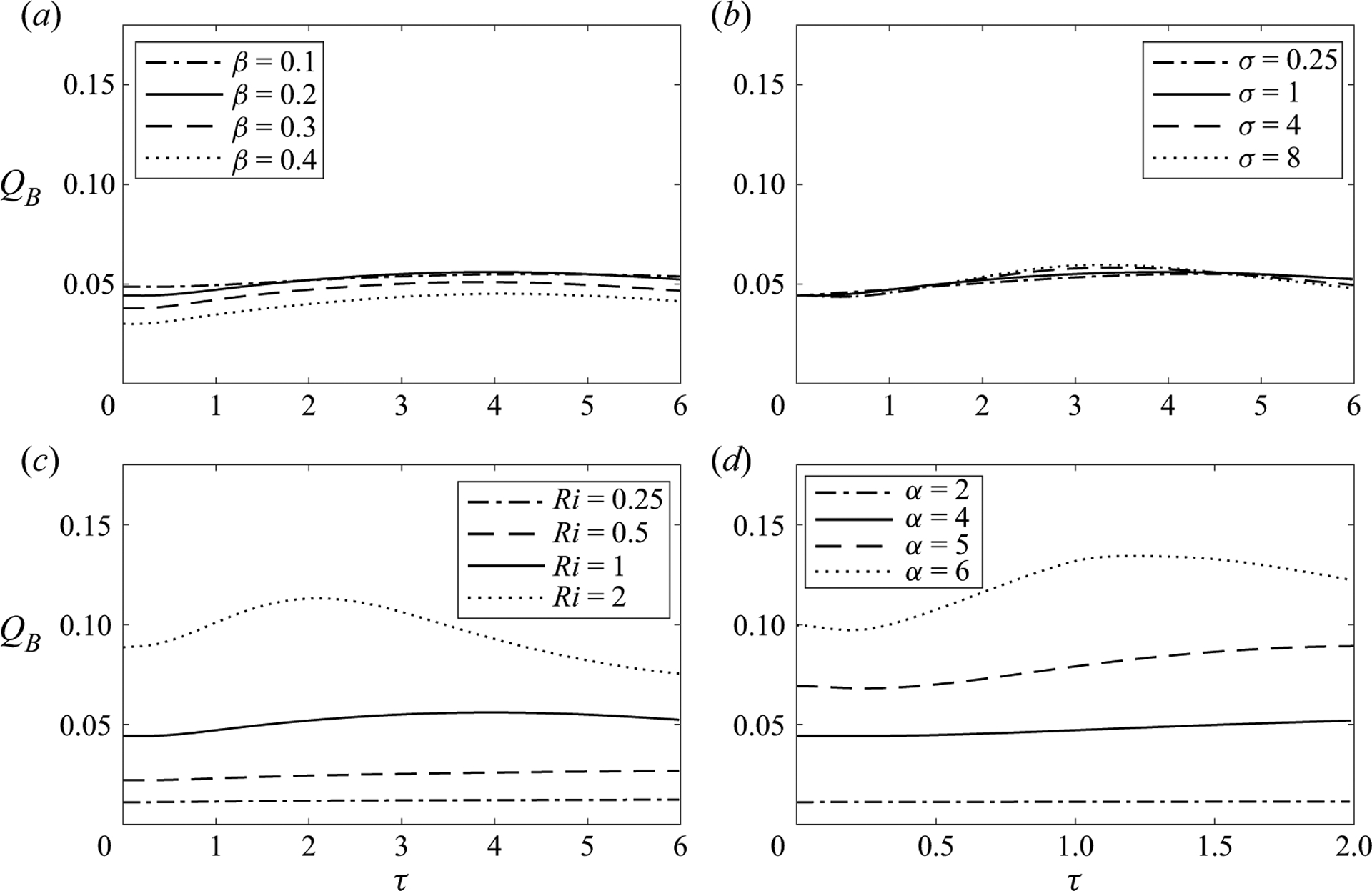
Influence of (*a*) the contraction ratio β, (*b*) reduced Schmidt number σ, (*c*) Richardson number Ri and (*d*) Womersley number α on the temporal evolution of the buoyancy-induced flow rate $Q_{B}$. The values of the parameters in each case are: (*a*) α=4,σ=Ri=1;(b)α=4,Ri=1 and β=0.2;(c)α=4,σ=1 and β=0.2;(d)σ=Ri=1 and β=0.2.

As in the buoyancy-free flow depicted in figure 3, the solution in figure 4 includes Lagrangian streamlines, colour contours of solute concentration, and streamwise distributions of integrated solute concentration $C=\int_{0}^{H}c$ dy along the canal. Buoyancy has a dramatic effect on the dispersion of the solute, as is apparent by comparing the results in both figures. Gravitational forces acting on the light solute induce a longitudinal pressure gradient that modifies drastically the resulting Lagrangian drift, as can be seen by comparing the streamlines in figure 3 with those in figure 4. The pattern of symmetric recirculating cells with unconnected streamlines existing for Ri=0 is replaced for Ri=1 by a more complicated streamline pattern featuring a net upward flow rate $Q_{B}(\tau )$ (see the solid curves in figure 5, to be discussed later). As can be seen, although the flow rate and the associated streamline pattern vary slowly in time, the observed changes are not very pronounced. Also of interest is that the quantitative agreement between the streamlines predicted by the model and the DNS results is again remarkable, thereby giving additional confidence in our development.

The changes in the Lagrangian motion have a dramatic reflection in the solute dispersion. As shown in figure 4, the solute is transported upwards following the Lagrangian streamlines connecting the cells, enabling its upward progression. The bolus of solute is distorted by the recirculating flow as it travels upwards, driven by the buoyancy-induced draft. The variation with time of the concentration distribution predicted with the model is in excellent agreement with the DNS results. The model is shown to predict not only the mean location of the bolus but also its shape and elongation. The relative departures, measured by $\left(\int_{0}^{n}\left|C_{DNS}-C_{\text{MODEL}}\right|dx\right)/\left(\int_{0}^{n}C_{i}\;dx\right)$, remain below 0.009 over the entire range of times shown in the figure. In assessing the potential benefits of the time-averaged formulation, it is important to emphasise that, although the integration of the integro-differential equation (4.8) over times τ~1 can be completed in a few hours using a laptop computer, generating the DNS results shown in figure 4, spanning over 3000 cardiac cycles, required 10 days in a computational cluster using a total of 72 cores.

### 5.4. Parametric dependence of the results

**Figure 6.**
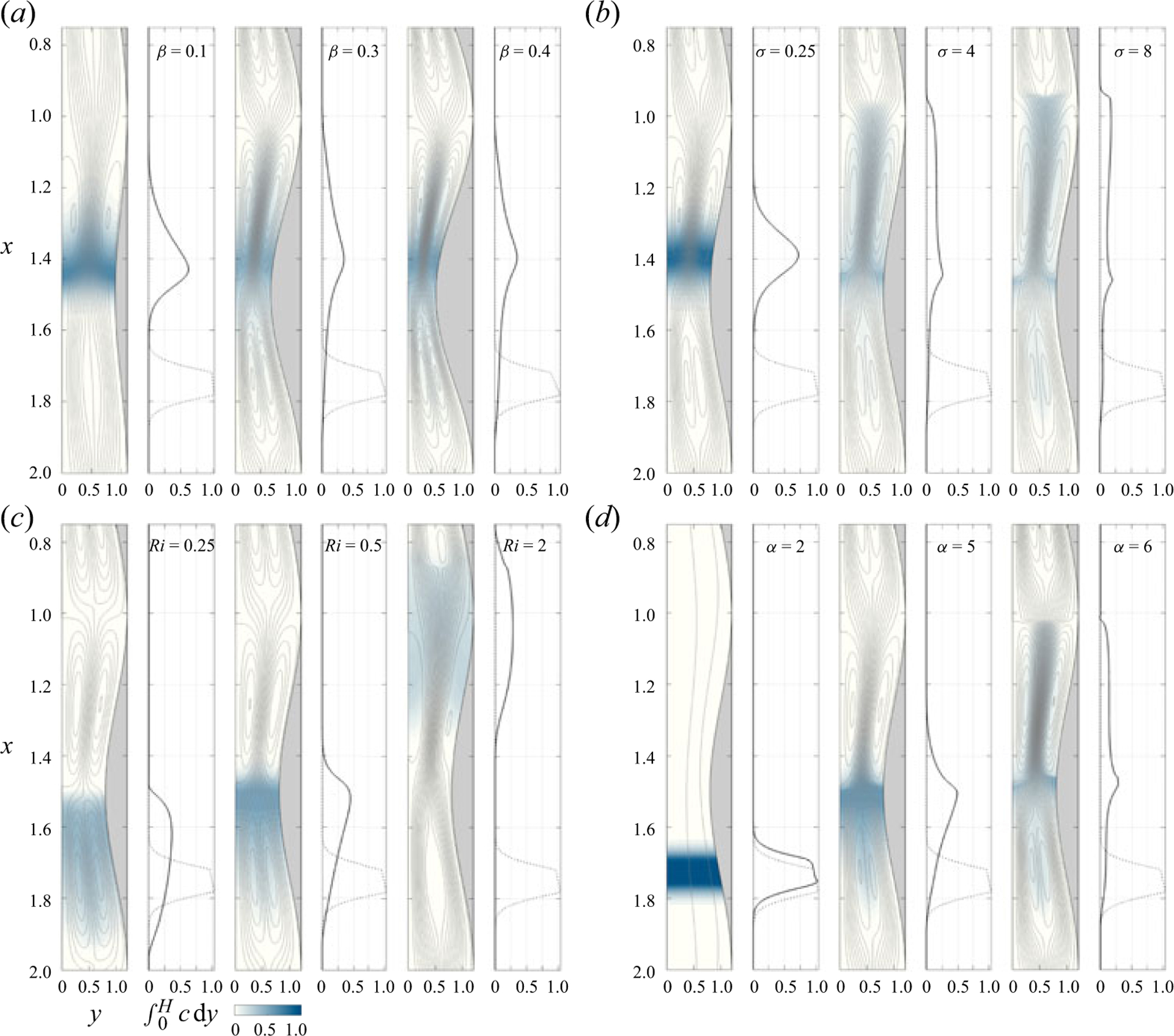
Influence of (*a*) the contraction ratio β, (*b*) reduced Schmidt number σ, (*c*) Richardson number Ri and (*d*) Womersley number α on the solute-concentration distribution and associated Lagrangian streamlines. The snapshots are taken at τ=6 for (a−c) and at τ=2 for (*d*). The values of the parameters in each case are: (a) α=4,σ=Ri=1; (*b*) α=4,Ri=1 and β=0.2; (*c*) α=4,σ=1 and β=0.2; (*d*) σ=Ri=1 and β=0.2. The streamline spacing for the mean Lagrangian velocity is δψ=0.003 for (*a-c*) and δψ=0.005 for (*d*).

The case shown in figure 4 , corresponding to β=0.2,α=4,σ=Ri=1 is used in figures 5 and 6 as a basis to investigate the influence of the different parameters on the dispersion of a buoyant solute. To that end, results are generated with use of (4.8) by modifying one of the four controlling parameters at a time, while keeping the other three fixed at the values selected earlier. Figure 5 shows the variation with time of the buoyancy-induced flow rate $Q_{B}$, whereas figure 6 shows instantaneous solute-concentration distribution and associated Lagrangian streamlines at a fixed time, namely, τ=(6,6,6,2) for figures 6(a)–6(d), with corresponding results for the base case at these times shown in two of the subpanels of figure 4.

We begin by discussing the effect of the channel geometry. As shown in figure 6(a), increasing the undulation of the channel from β=0.1 to β=0.4 tends to increase the magnitude of the buoyancy-induced longitudinal velocity in the region of minimum cross-sectional area, where streamlines are closely spaced together for larger β, but these changes result in only moderately small variations of the flow rate $Q_{B}$, as shown in figure 5(a). As a result, the bolus of solute becomes more elongated as β increases, but advances upward at approximately the same rate, so that the maximum concentration occupies approximately the same location at τ=6, as shown in figure 6(a).

The effect of the Schmidt number σ, entering in the formulation only through the factor affecting the transverse diffusion rate on the right-hand side of (4.8), is investigated in figures 5(b) and 6(b). The changes observed in streamline pattern and flow rate when changing the Schmidt number from σ=0.25 to σ=8 are not very significant, so that the differences in solute evolution in figure 6(b) are attributable to the direct effect of transverse diffusion (or lack thereof). The snapshot corresponding to σ=8 displays the behaviour expected at large Schmidt numbers, for which fluid particles maintain a nearly constant concentration in their slow Lagrangian evolution, as described by the limiting form of (4.8) for σ≫1. In the opposite limit σ≪1, solute diffusion leads to a rapid uniformisation of the concentration, as can be seen by integrating $\partial^{2}c/\partial y^{2}=0$ (i.e. the reduced form of (4.8) when σ≪1) subject to the non-permeability condition ∂c/∂y=0 at y=0,H to give c=c(x,τ). As a result, the bolus remains relatively compact as it moves along the channel with a velocity determined by the flow rate. The reduced transport equation governing the transport of solutes with σ≪1 can be derived from (4.9) by noting that in this limit the integrated solute concentration $C=\int_{0}^{H}c\;dy$ becomes C=H(x)c(x,τ) while the solute flux $\phi (x,\tau )=\int_{0}^{H}\left(u_{SD}+u_{SS}+u_{B}\right)c$ dy reduces to $\phi =Q_{B}c$. Substituting these simplified expressions into (4.9) and using (3.29) to evaluate $Q_{B}$ finally yields

(5.2) $$\frac{\partial c}{\partial \tau }+\frac{\alpha^{2}Ri\int_{0}^{n}c\;dx}{12nH\int_{0}^{1}H^{-3}\;dx}\frac{\partial c}{\partial x}=0$$

as the limiting form of (4.8) for σ≪1

As is to be expected from the velocity expressions derived in §3.4, the Richardson number and the Womersley number have a pronounced effect on the mean Lagrangian motion. As shown in figures 5(c) and 5(d), the flow rate exhibits dependences on Ri and α that are approximately linear and approximately quadratic, respectively, consistent with (3.29). These dependences have a reflection on the evolution of the solute bolus shown in figures 6(c) and 6(d). With limited updraft for Ri=0.25, the bolus is seen to spread about the injection location, without significant upward progression at the instant of time τ=6 considered in the figure. An increase in Ri promotes the displacement of the bolus, but its longitudinal extent remains approximately equal in all three cases. By way of contrast, an increase in α increases the upward displacement and also enhances bolus distortion. The reason for the latter is that larger values of α hinder transverse diffusion, as can be inferred from (4.8), with the result that fluid particles travel following the Lagrangian recirculating paths with a nearly constant concentration, rapidly deforming the compact concentration distribution of the initial bolus.

## 6. Conclusions

Solute dispersion in a wavy-walled vertical channel subject to an oscillating pressure gradient has been used as a canonical model to investigate the effect of buoyancy on the transport of ITDD drugs, characterised by large values of the Schmidt number and order-unity values of the Richardson number. The mean Lagrangian velocity determined in the asymptotic limit of small stroke lengths, responsible for the convective transport of the solute, displays a buoyancy component whose local value depends on the solute concentration through integral expressions, resulting in a nonlinear integro-differential transport equation. The predictive capabilities of the reduced description are tested through comparisons with DNS computations involving thousands of oscillating cycles. The validation exercise reveals that the model provides accurate predictions of solute dispersion at a fraction of the computational cost involved in the DNS. In contrast to the motion observed in the buoyancy-free case investigated in figures 2 and 3, characterised by the existence of a series of closed Lagrangian vortices distributed periodically along the channel, the buoyancy-modulated mean Lagrangian flow shown in figure 4 includes a streamwise draft connecting neighbouring cells that promotes the longitudinal dispersion of the solute. The buoyancy-enhanced transport rate revealed in our channel computations is consistent with previous clinical observations pertaining to dispersion of light drugs for patients in a sitting injection position (Richardson *et al*. 1996).

The simple canonical flow considered here has served to unveil some of the key aspects of the solute-dispersion problem, including the enhanced transport associated with the buoyancy-modulated mean Lagrangian velocity. Future work should consider application of the two-time-scale asymptotic analysis delineated previously to the description of ITDD processes, with account taken of the three-dimensional morphology of the spinal canal, possibly including the effect of microanatomical features such as arachnoid trabeculae, which are thin strands of connective tissue that form a web-like structure stretching across the spinal canal. The presence of these fine anatomical structures, which has been shown to have an important effect on pressure loss (Tangen *et al*. 2015), can be accounted for by treating the spinal subarachnoid space as a Brinkman porous medium, as done in previous investigations (Gupta *et al*. 2009; Kurtcuoglu, Jain & Martin 2019; Salerno, Cardillo & Camporeale 2020; Sincomb *et al*. 2022).

The future developments envisioned here can potentially provide a reduced transport equation, possibly similar to (4.8), to be used in combination with magnetic resonance imaging characterisations of the canal anatomy (Coenen *et al*. 2019) to describe the transport of the drug in the relevant dispersion time scale. The ultimate goal of such efforts is the development of computationally effective subject-specific predictive tools for drug delivery to a target site from injection by a lumbar puncture with account taken of the specific anatomy and physiological conditions of the individual patient as well as for the molecular characteristics and injection rate of the drug, as needed in guiding clinical treatments.
